# Detection of Central Retinal Artery Occlusion by Point-of-Care Ultrasound in the Emergency Department: A Case Series

**DOI:** 10.7759/cureus.16142

**Published:** 2021-07-03

**Authors:** Kevin R Caja, Kaylan M Griffith, Kevin R Roth, Charles C Worrilow, Marna R Greenberg, Theodore B Doherty

**Affiliations:** 1 Department of Emergency and Hospital Medicine, Lehigh Valley Health Network, Allentown, USA; 2 Department of Emergency Medicine, University of South Florida (USF) Morsani College of Medicine/Lehigh Valley Health Network Campus, Allentown, USA

**Keywords:** pocus, crao

## Abstract

Central retinal artery occlusion (CRAO) is a rare, but serious, diagnosis that can lead to blindness, most often due to thromboembolic disease. In the emergency department (ED), CRAO can present as acute, painless loss of vision. Physicians need quick ways to rule in this diagnosis due to the time-sensitive nature of the event. We describe two patients in this cases series who present to the same ED with unilateral painless vision loss and histories that include notable risk factors such as thromboembolic and atherosclerotic disease. Upon arrival, point-of-care ultrasound (POCUS) done at the bedside allowed for quick determination of CRAO. ​​​​​​​The importance of this case series is to emphasize the efficacy of POCUS in evaluating patients with painless vision loss in the ED setting.

## Introduction

Central retinal artery occlusion (CRAO) is a rare, but vision-threatening event that requires prompt diagnosis [1]. Risk factors include hypertension, diabetes, atherosclerotic disease, and smoking with greater incidence in men and with increasing age [2]. Standard diagnosis relies on visual acuity testing, relative afferent pupillary defect evaluation, fundoscopic exam, and fluorescein angiography [1,2]. These methods, although effective, are time-consuming, resource-dependent, and many times ophthalmologist-dependent [3]. Considering ocular emergencies can be up to 3% of ED visits, point-of-care ultrasound (POCUS) has emerged as a staple in this work-up due to its speed, ease of use, and reliability [3]. Presented here are two cases of CRAO evaluated by POCUS and recognized by the “spot sign”, a hyperechoic object within the central retinal artery, likely a clot, behind which a hypoechoic edematous optic nerve can be visualized [4]. 

## Case presentation

Case 1

**Figure 1:**
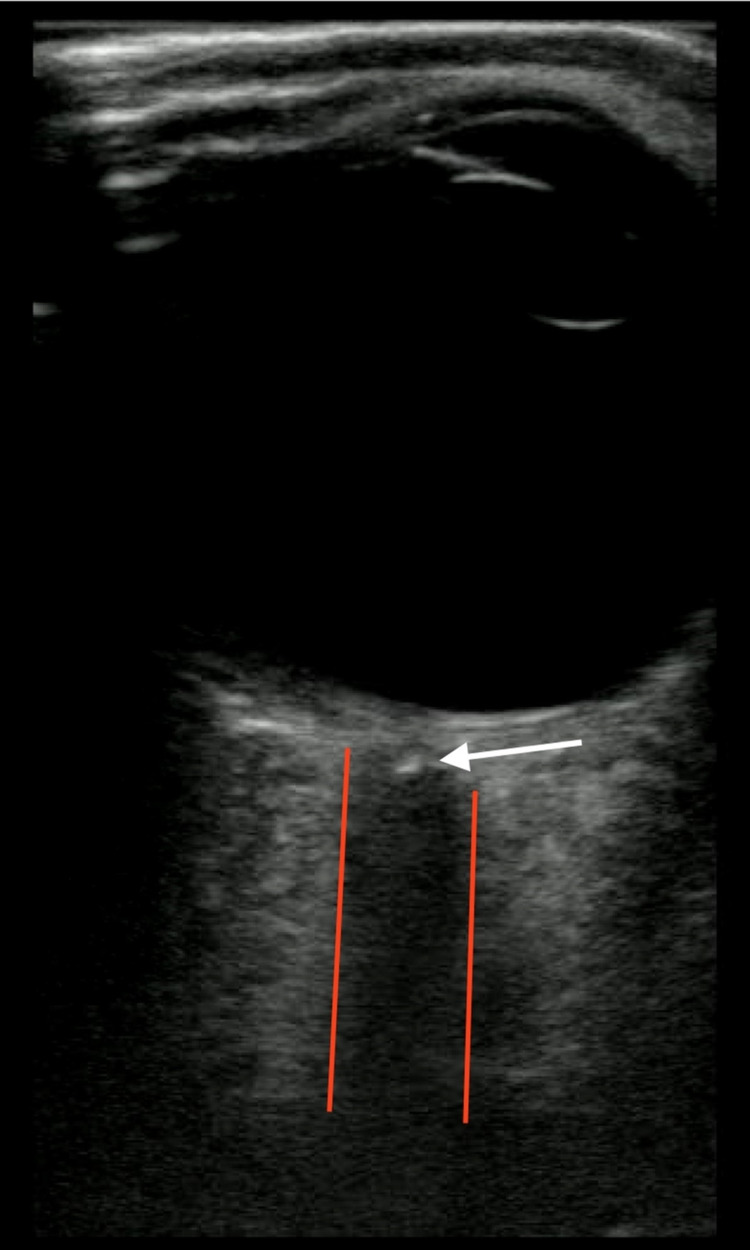
Ocular point-of-care ultrasound showing "spot sign".

A 65-year-old male presented to the ED after acute painless loss of vision in his right eye. He reported black spots starting the previous evening and then that same evening around 9:30 pm it was as if the “lights were turned out”. He believed this would get better with sleep; however, when he woke up he still could not see anything with his right eye. He was not on any blood thinners. He was an active smoker with a history of hypertension and hyperlipidemia. He denied headache, chest pain, cough, shortness of breath, abdominal pain, nausea, vomiting, or diarrhea. Visual acuity was 20/light perception centrally in the right eye (OD) and 20/20 with glasses in the left eye (OS). Intraocular pressures were both 20mm. His right pupil was 6 mm and minimally reactive to light and his left pupil was 4mm, and reactive with a 2+APD (afferent pupillary defect). POCUS showed an obvious “Spot Sign” on the right eye (Figure 1). Ophthalmology was immediately contacted and agreed that the patient would need to be admitted for a stroke/embolism workup. MRI/MRA of the head and neck were ordered in the ED and the patient was admitted. The MRI/MRA had no pertinent findings. The patient was started on atorvastatin and clopidogrel and continued on all his previous medications.

Case 2

**Figure 2:**
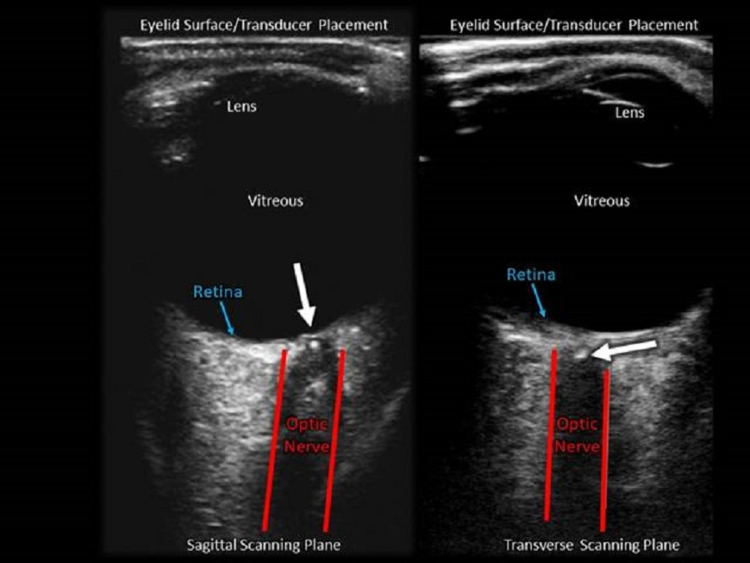
Sagittal and transverse scanning plane in an ocular point-of-care ultrasound showing "spot sign".

A 76-year-old female presented to the ED after acute painless vision loss in her left eye. She awoke with severely blurred vision and constricted peripheral vision of the affected eye. The patient had a significant thromboembolic history including pulmonary embolism, myocardial infarction, and middle cerebral artery (MCA) territory infarction followed by recurrent ischemia. Visual acuity was 20/30 in the right eye (OD), and only hand motion detectable in the left eye (OS). Intraocular pressures were both 13mm. Neurologic and standard external eye exams were otherwise unremarkable. POCUS immediately recognized the classic “Spot sign” (Figure 2) and ophthalmology was contacted. The ophthalmologist chose to confirm the diagnosis via a dilated eye fundoscopic examination in the ED. CT Angiogram followed by an MRI of the brain did not show any acute neurologic infarction. Since the patient was already anticoagulated on Apixaban for prior strokes, no additional interventions were advised by specialists. Unfortunately, the patient’s vision has not returned. 

## Discussion

These two cases of CRAO, an ocular emergency, showcase the utility of POCUS in allowing for rapid diagnosis, prompt ophthalmologic consult, and hopefully decreasing time to definitive care. The classic “spot sign” can distinguish CRAO from other causes of unilateral painless vision loss such as Giant Cell (Temporal) Arteritis or retinal detachment [2,4]. Rapid identification of a thrombus is key to prevent long-term disability through the use of interventions such as ocular massage, thrombolysis, and reduction of IOP through surgery [2,4-7]. It also allows earlier initiation of stroke work-up [4,6,7]. Various cases have been described that similarly point to the efficacy of POCUS as a diagnostic tool for emergency physicians [1,6,8-10]. It is efficient, portable, non-invasive, and well-tolerated [10,11]. Prior to the integration of POCUS work-up of CRAO consisted mainly of direct fundoscopy; this required direct visualization of the intraocular structures and pupillary dilation [1]. This is potentially concerning since the skills to detect abnormalities reliably by fundoscopy performed by ophthalmologists and non-ophthalmologists are reportedly declining [12]. Other standard of care tests include visual acuity testing, evaluation for afferent pupillary defect, slit-lamp examination, and fluorescein angiography and ophthalmology consultation [1,2]. POCUS has been shown to be an effective tool for diagnosing other ocular emergencies, like retinal detachment, vitreous hemorrhage, and vitreous attachment [13,14]. Ocular POCUS is useful for evaluating ocular complaints presenting to the ED, and physicians should feel confident using this modality [14]. As in our cases, variability exists between the confidence practitioners might have in POCUS. It is therefore vital to continue publishing on the use of POCUS, in the diagnosis of CRAO, to raise awareness among the medical community and eliminate this variability. Albeit small studies, the sensitivity (83%) and specificity (100%) as well as the strong interobserver agreement (Cohen’s kappa 0.98) of POCUS for CRAO are worthy of our confidence in this tool [15,16]. Authors concede that it is not yet the gold standard for this diagnosis because it remains unknown what percentage of patients with a confirmed CRAO by fundus exam and retinal angiography have this ultrasound finding. However, the findings demonstrated in our cases are very helpful for ED management. These outcomes and the use of POCUS are likely to continue to increase as instrument quality and user education improves. Institutions should disseminate what information can reliably obtain through POCUS. The proliferation of electronic archiving allows real-time review by consultants of those images captured by physicians who are at the patient’s bedside. These elements should lead to a collegial and comprehensive approach to patient care. In our situation, the two cases presented to the ED within 24 hours and both were quickly identified with POCUS. 

## Conclusions

Due to the rarity and severity of central retinal artery occlusion, it is important for physicians to quickly diagnose patients. This case highlights how point-of-care ultrasounds can allow for rapid diagnosis, prompt ophthalmologic consult, and hopefully decrease time to definitive care. As utilization and their familiarity increase, the use of bedside ultrasound should improve the transition of care to the ophthalmologist and allow for quick and accurate evaluation of time-sensitive conditions.
